# UNDERSTANDING OLDER HEALTH CARE WORKERS’ BURNOUT DURING COVID-19

**DOI:** 10.1093/geroni/igac059.1792

**Published:** 2022-12-20

**Authors:** Jihee Woo, Rafael Engel

**Affiliations:** University of Minnesota, St. Paul, Minnesota, United States; University of Pittsburgh, Pittsburgh, Pennsylvania, United States

## Abstract

The prolonged battle against the COVID-19 pandemic has left many health care workers physically, mentally, and emotionally exhausted, exacerbating burnout that was already endemic in the healthcare sector. Burnout places not only an individual's well-being but patient care at risk. In this study, we examine protective and risk factors contributing to burnout specifically among older health care workers during COVID-19. We address these questions using data collected from an online survey conducted on health care workers employed at hospitals in Pittsburgh, Pennsylvania in February 2022. Health care workers ages 50 and older (n=165) –a subset of surveyed health care workers– were included in the analytic sample. Participants were asked a series of questions about burnout, mental health, workplace stressors, intent to leave the job, and demographics. Participants were predominantly female (88%) and white (91%) with a mean age of 57.2 years. Almost 70% were registered nurses and the remaining were service, clerical, and technical workers; the mean years of work experience was 21.5 years. Most participants (77%) experienced moderate levels of burnout. Preliminary regression analysis suggests that perceived inadequate staffing (β=2.12, p<.001) and workplace discrimination (β=1.23, p=.001) were positively associated with burnout while job autonomy (β=-1.77, p<.05) and schedule flexibility (β=-.2.09, p=.001) were negatively associated with burnout. Burnout was in turn positively associated with depression, anxiety, and intent to leave the job. These findings demonstrate the need for workplace support to address older health care workers’ burnout, better accommodate their needs, and keep them safe and healthy in their jobs.

